# Efficacy of probiotics and fibers on metabolic disturbances associated with antipsychotics: A systematic review and network meta-analysis

**DOI:** 10.1192/j.eurpsy.2023.686

**Published:** 2023-07-19

**Authors:** Y. Soliman, N. I. Hendi, J. Magdy Daniel, M. Emara, N. M. Gharib, O. A. Abdelwahab, R. U. Awan

**Affiliations:** ^1^Faculty of Medicine, Assiut University, Assiut; ^2^Faculty of Medicine, Ain Shams University, Cairo; ^3^Faculty of Medicine, Menofia University, Menofia; ^4^Faculty of Medicine, Port Said University, Port Said; ^5^Faculty of Medicine, Alexandria University, Alexandria; ^6^Faculty of Medicine, Al-Azhar University, Cairo, Egypt; ^7^Ochsner Health System, Mississippi, United States

## Abstract

**Introduction:**

Human gut microbiota plays an important role in metabolic health. Atypical antipsychotics can lead to metabolic abnormalities and changes in the gut microbiota. Multiple studies have examined the role of probiotics in suppressing antipsychotics-induced weight gain, but they have never been examined in a meta-analysis.

**Objectives:**

This network meta-analysis aims to compare the effect of probiotics + fibers, probiotics only, and fibers only on metabolic abnormalities induced by atypical antipsychotics.

**Methods:**

We searched PubMed, Scopus, Web of Science, and Cochrane Controlled Register of Trials (CENTRAL) for all relevant studies. We used mean difference with its 95% confidence interval as our effect size. Primary outcomes were body weight and body mass index (BMI), while secondary outcomes were changes in other cardiometabolic risk factors.

**Results:**

We included 4 randomized controlled trials comprising 319 patients. For body weight, probiotics + fibers (MD -3.96, 95% CI [-5.16, -2.76]), fibers only (MD -1.91, 95% CI [-3.81, -0.01]), and probiotics only (MD -1.37, 95% CI [-2.07, 0.66]) were significantly superior to placebo. Probiotics + fibers (MD -1.52, 95% CI [-2.11, -0.92]), but not fibers only or probiotics only, was associated with significant changes in BMI. Probiotics + fibers was also associated with significant changes in cholesterol (MD -0.37, 95% CI [-0.67, -0.07]), insulin levels (MD -3.37, 95% CI [-5.35, -2.10]), and insulin resistance index (MD -1.35, 95% CI [-1.94, -0.76]). There were no significant adverse events reported in the included studies.

**Conclusions:**

Probiotics + fibers, probiotics only, and fibers can be effective in controlling antipsychotics-induced metabolic abnormalities, with probiotics + fibers being the most effective regimen. All three treatments were safe and well tolerated by patients.

**Disclosure of Interest:**

None Declared

